# Efficacy and safety of Qi and Blood Tonic Chinese Medicines in the treatment of COVID-19: A protocol for systematic review and meta-analysis

**DOI:** 10.1097/MD.0000000000032136

**Published:** 2022-12-09

**Authors:** Feifei Yang, Xiaosi Zhang, Zhe Zhang, Hao Lu, Jiawei Li, Ning Bai, Naizhi Geng

**Affiliations:** a Heilongjiang University of Traditional Chinese Medicine, Harbin, China; b Beijing University of Traditional Chinese Medicine, Beijing, China; c Department of Traditional Chinese Medicine, affiliated Hospital of North Sichuan Medical College, Nanchong, China; d Tianshui Hospital of Traditional Chinese Medicine, Tianshui, China; e First Hospital of Heilongjiang University of Chinese Medicine, Harbin, China.

**Keywords:** COVID-19, meta-analysis, protocol, Qi and blood tonify Chinese medicines, systematic review, TCM

## Abstract

Coronavirus disease in 2019 (COVID-19) is a sudden public event affecting all human beings, with the rapid transmission, extensive groups affected, many complications, and high mortality. Traditional Chinese Medicine has a long history of preventing and treating infectious diseases, and numerous studies have shown that Traditional Chinese Medicine, especially herbal medicine, has a positive effect on the prevention, treatment, and post-healing recovery of this COVID-19, and herbal medicines to supplement qi and blood often occupy a certain proportion of it. However, there is no relevant meta-analysis to date. Therefore, this study aims to evaluate the efficacy and safety of qi and blood tonic herbal medicines in the treatment of COVID-19 through Systematic Review and meta-analysis to provide a reference basis for widespread clinical application.

**Methods:**

We will search from the following databases for the period from the time of database construction to March 1st, 2023. The English databases include: PubMed, MEDLINE, EMBASE, Cochrane library, WOS, Google Scholar, and CENTRAL; The Chinese databases include: China National Knowledge Infrastructure, China Biomedical Literature Database, Technology Journal Database, and Wanfang. Randomized controlled trials in English or Chinese that include Chinese herbal medicines for tonifying Qi and Blood in the treatment of patients with COVID-19 will be included. Data were independently screened and collected by 2 investigators. The risk of bias for each trial was assessed using the Cochrane Risk of Bias Tool 2.0. RevMan 5.3 software was used for the meta-analysis of the data. Primary outcome indicators included cure, mortality, and exacerbation rates (change in disease severity category, patient admission to ICU, etc.). Secondary outcome indicators included recovery rate or duration of major symptoms (e.g., fever, cough, fatigue, and weakness, etc.), rate or duration of nucleic acid conversion for severe acute respiratory syndrome coronavirus-2, improvement or recovery of chest CT performance, length of hospital stay, and other adverse events.

**Results:**

This protocol adheres to the Preferred Reporting Items for Systematic Reviews and Meta-Analyses-P guidelines to ensure clarity and completeness of reporting in all phases of the systematic review.

**Conclusion:**

This study will provide evidence regarding the efficacy and safety of Qi and Blood Tonic Chinese Medicines for the treatment of COVID-19.

**PROSPERO registration number:**

CRD42022361822 (https://www.crd.york.ac.uk/prospero/display_record.php?ID=CRD42022361822).

**Strengths and limitations of this study**

• The purpose of this protocol is to evaluate the efficacy and safety of Chinese herbal medicines that tonifies Qi and Blood in the treatment of COVID-19. In addition, the effect of different interventions on COVID-19 can be assessed by subgroup analysis.
• Our accurate assessment may provide a new solution idea for the treatment and rehabilitation of COVID-19 patients, which may help clinicians to diversify their treatment modalities. In addition, the data in this study protocol were collected and analyzed independently by at least 2 investigators.
• It is not clear to us whether all patients had better adherence and whether there were uncontrolled or unmeasured confounders that may have biased the results.
• It is also unclear to us whether the included studies differentiated between disease populations (e.g., age, sex, region), which may have produced some selection bias
• Differences in the doses of herbal medicines in the selected formulas may increase the risk of heterogeneity.

## 1. Introduction

In January 2020, a newly discovered coronavirus in Wuhan, Hubei Province, China, was named the novel coronavirus “2019-nCoV.”^[1]^ The disease caused by this virus was subsequently identified as COVID-19 (the full name “coronavirus disease in 2019”).^[2]^ As the number of confirmed cases and deaths in China and other countries surged, on March 11, 2020, the World Health Organization officially characterized COVID-19 as a pandemic sweeping the world.^[3]^ COVID-19 not only includes common symptoms such as dry cough, fever, dyspnea, myalgia, and headache,^[4–6]^ but also causes or aggravates, for example, pathologies in the central nervous system,^[7–9]^ gastrointestinal tract,^[10]^ heart, blood, liver, kidneys, and numerous other organs and tissues.^[11–14]^ In addition, the clinical manifestations of novel coronavirus pneumonia are more pronounced in patients aged over 60 years,^[15]^ whose treatment is more difficult, with a longer course and poorer healing, and therefore treatment and care of older adults over 60 years should be given more attention. To date, the virus is still spreading in many countries, including China, and as of October 16, 2022, the cumulative number of confirmed cases worldwide exceeds 621 million, with more than 6.5 million deaths.^[16]^ And this number is showing a continuous increase as reported by various organizations.

In China, Traditional Chinese Medicine (TCM) has become a consensus treatment for novel coronavirus disease,^[17]^ from the third edition^[18]^ to the ninth edition^[19]^ of the treatment protocol for pneumonia with novel coronavirus infection jointly issued by the National Health and Wellness Commission of China and the State Administration of Traditional Chinese Medicine. In addition, TCM is gaining popularity overseas.^[20,21]^ Early intervention and full involvement of TCM can effectively prevent the mild disease from turning into severe disease and alleviate symptoms, thus greatly improving the cure rate and shortening the course of the disease, and reducing mortality.^[22,23]^ For example, the treatment of new coronavirus pneumonia with Lianhua Qingwen in combination with western medicine can greatly improve the overall efficiency and TCM comprehensive symptom score, and the involvement of Xuebijing injection can significantly reduce interleukin-6 (IL-6) levels and body temperature.^[22]^ A retrospective analysis showed that for patients recovering from neocoronary pneumonia, TCM involvement in treatment resulted in significant improvements including inflammation-related indicators such as white blood cell count and serum IL-6, and was beneficial for improving cardiac and liver function.^[24]^

From ancient times to the present, Chinese medicine has emphasized the protection of vital energy in the prevention and treatment of infectious diseases.^[25]^ In this novel coronavirus disease, as an important way to replenish the vital energy of the qi, tonifying herbs such as Huangqi, Renshen, Dangshen, Xiyangshen, Baizhu, Shanyao, Gancao, Danggui, Shudi, Hongjingtian, Baishao, Gouqi, Dazao have been occupied a special place in the formulas.^[26,27]^

## 2. Materials and Methods

The protocol is registered with PROSPERO under registration number CRD42022361822 and was prepared in accordance with the Preferred Reporting Items for Systematic Review and Meta-Analysis Protocol guidelines.^[28]^

### 2.1. Criteria for inclusion in the study

#### 2.1.1. Types of studies.

Studies of randomized controlled trials (RCTs) of treatment of COVID-19 with herbs that tonify Qi and Blood will be included. No restrictions on publication language or publication status. Both published and unpublished RCTs will be included, but non-randomized trials will not be included.

#### 2.1.2. Types of participants.

Patients diagnosed with COVID-19 will be enrolled, with no restrictions on gender, age, nationality, or race.

#### 2.1.3. Types of intervention.

The intervention for the test group should be a prescription containing Chinese herbal medicine to tonify qi and blood (including Chinese herbal medicine and proprietary Chinese medicine) alone, or the prescription in combination with other interventions. The control group can use any other treatment method other than the prescription of Chinese herbal medicine for tonifying Qi and Blood (e.g., prescription of Chinese herbal medicine without tonifying Qi and Blood, Western medicine treatment, Chinese and Western external medicine, conventional or standard care, placebo or no treatment, etc.).

#### 2.1.4. Types of outcome.

Primary outcome indicators included cure rate, mortality rate, and exacerbation rate (change in disease severity category, or patient admission to ICU, etc.). Secondary outcome indicators included recovery rate or duration of major symptoms (e.g., fever, cough, fatigue, weakness, etc.), rate or duration of nucleic acid regression for severe acute respiratory syndrome coronavirus-2 (SARS-CoV-2), improvement or recovery of chest CT performance, length of hospital stay, and other adverse events.

### 2.2. Information sources

We will conduct literature searches in PubMed, Excerpta Medica database, Cochrane Library, Web of Science, Google Scholar, China National Knowledge Infrastructure, China Biomedical Literature Database, China Science and Technology Journal Database, and Wanfang Database (Wanfang). In addition, unpublished data from ongoing trials will be retrieved from the China Clinical Trials Registry (ChiCTR) and ClinicalTrials.gov (www.ClinicalTrials.gov/). Searches of all databases and online registry platforms will be available from the time of build until March 1st, 2023, with no language restrictions. Searches will be conducted using subject terms plus titles or abstracts; Chinese searches will use Chinese translations of the search terms. The full process of searching on PubMed is detailed in Table 1.

**Table 1:** PubMed example of literature search strategy.

Search number	Query
#1	Qi OR Blood Tonic Chinese Medicines[MeSH Terms]
#2	Qi[Title/Abstract] OR Blood Tonic Chinese Medicines[Title/Abstract]
#3	renshen or huangqi or baizhu or shanyao or gancao or dangshen or hongshen or xiyangshen or taizishen or ziheche or longyanrou or dazao or yitang or jingmi or hongjingtian or zheshizi or ciwujia or baibiandou or lingzhi or gaolishen or turenshen tushen panlongshen zhujieshen or jiaogulan or wuzhimao[MeSH Terms]
#4	renshen[Title/Abstract] OR huangqi[Title/Abstract] OR baizhu[Title/Abstract] OR shanyao[Title/Abstract] OR gancao[Title/Abstract] OR dangshen[Title/Abstract] OR hongshen[Title/Abstract] OR xiyangshen[Title/Abstract] OR taizishen[Title/Abstract] OR ziheche[Title/Abstract] OR longyanrou[Title/Abstract] OR dazao[Title/Abstract] OR yitang[Title/Abstract] OR jingmi[Title/Abstract] OR hongjingtian[Title/Abstract] OR zheshizi[Title/Abstract] OR ciwujia[Title/Abstract] OR baibiandou[Title/Abstract] OR lingzhi[Title/Abstract] OR gaolishen[Title/Abstract] OR turenshen[Title/Abstract] OR tushen[Title/Abstract] OR panlongshen[Title/Abstract] OR zhujieshen[Title/Abstract] OR jiaogulan[Title/Abstract] OR wuzhimao[Title/Abstract]
#5	honey[MeSH Terms]
#6	honey[Title/Abstract]
#7	shudihuang or danggui or baishao or shouwu or ejiao or haishen or heizhima or sangshen[MeSH Terms]
#8	shudihuang[Title/Abstract] OR danggui[Title/Abstract] OR baishao[Title/Abstract] OR shouwu[Title/Abstract] OR ejiao[Title/Abstract] OR haishen[Title/Abstract] OR heizhima[Title/Abstract] OR sangshen[Title/Abstract]
#9	#1 or #2 or #3 or #4 or #5 or #6 or #7 or #8
#10	(COVID-19 or COVID Virus Disease or COVID-19 Virus Disease or COVID Virus Diseases or Disease, COVID Virus or Virus Disease, COVID or COVID Virus Infection or COVID-19 Virus Infection or COVID Virus Infections or Infection, COVID Virus or Virus Infection, COVID or 2019-nCoV Infection or 2019 nCoV Infection or 2019-nCoV Infections or Infection, 2019-nCoV or Coronavirus Disease-19 or Coronavirus Disease 19 or 2019 Novel Coronavirus Disease or 2019 Novel Coronavirus Infection or 2019-nCoV Disease or 2019 nCoV Disease or 2019-nCoV Diseases or Disease, 2019-nCoV or COVID or Coronavirus Disease 2019 or Disease 2019, Coronavirus or SARS Coronavirus 2 Infection or SARS-CoV-2 Infection or Infection, SARS-CoV-2 or SARS CoV 2 Infection or SARS-CoV-2 Infections or COVID Pandemic or COVID 19 Pandemic or COVID Pandemics or Pandemic, COVID) AND (COVID-19 or COVID Virus Disease or COVID-19 Virus Disease or COVID Virus Diseases or Disease, COVID Virus or Virus Disease, COVID or COVID Virus Infection or COVID-19 Virus Infection or COVID Virus Infections or Infection, COVID Virus or Virus Infection, COVID or 2019-nCoV Infection or 2019 nCoV Infection or 2019-nCoV Infections or Infection, 2019-nCoV or Coronavirus Disease-19 or Coronavirus Disease 19 or 2019 Novel Coronavirus Disease or 2019 Novel Coronavirus Infection or 2019-nCoV Disease or 2019 nCoV Disease or 2019-nCoV Diseases or Disease, 2019-nCoV or COVID or Coronavirus Disease 2019 or Disease 2019, Coronavirus or SARS Coronavirus 2 Infection or SARS-CoV-2 Infection or Infection, SARS-CoV-2 or SARS CoV 2 Infection or SARS-CoV-2 Infections or COVID Pandemic or COVID 19 Pandemic or COVID Pandemics or Pandemic, COVID[MeSH Terms])
#11	COVID-19[Title/Abstract] OR COVID Virus Disease[Title/Abstract] OR COVID-19 Virus Disease[Title/Abstract] OR COVID Virus Diseases[Title/Abstract] OR Disease, COVID Virus[Title/Abstract] OR Virus Disease, COVID[Title/Abstract] OR COVID Virus Infection[Title/Abstract] OR COVID-19 Virus Infection[Title/Abstract] OR COVID Virus Infections[Title/Abstract] OR Infection, COVID Virus[Title/Abstract] OR Virus Infection, COVID[Title/Abstract] OR 2019-nCoV Infection[Title/Abstract] OR 2019 nCoV Infection[Title/Abstract] OR 2019-nCoV Infections[Title/Abstract] OR Infection, 2019-nCoV[Title/Abstract] OR Coronavirus Disease-19[Title/Abstract] OR Coronavirus Disease 19[Title/Abstract] OR 2019 Novel Coronavirus Disease[Title/Abstract] OR 2019 Novel Coronavirus Infection[Title/Abstract] OR 2019-nCoV Disease[Title/Abstract] OR 2019 nCoV Disease[Title/Abstract] OR 2019-nCoV Diseases[Title/Abstract] OR Disease, 2019-nCoV[Title/Abstract] OR COVID[Title/Abstract] OR Coronavirus Disease 2019[Title/Abstract] OR Disease 2019, Coronavirus[Title/Abstract] OR SARS Coronavirus 2 Infection[Title/Abstract] OR SARS-CoV-2 Infection[Title/Abstract] OR Infection, SARS-CoV-2[Title/Abstract] OR SARS CoV 2 Infection[Title/Abstract] OR SARS-CoV-2 Infections[Title/Abstract] OR COVID Pandemic[Title/Abstract] OR COVID 19 Pandemic[Title/Abstract] OR COVID Pandemics[Title/Abstract] OR Pandemic, COVID[Title/Abstract]
#12	#10 or #11
#13	RCT or RCTs or Randomized controlled trials or Randomized controlled trial[MeSH Terms]
#14	RCT[Title/Abstract] OR RCTs[Title/Abstract] OR Randomized controlled trial[Title/Abstract] OR Randomized controlled trials[Title/Abstract]
#15	#13 or #14
#16	#9 and #12 and #15

### 2.3. Search strategy

#### 2.3.1. Database search.

**Figure 1.**
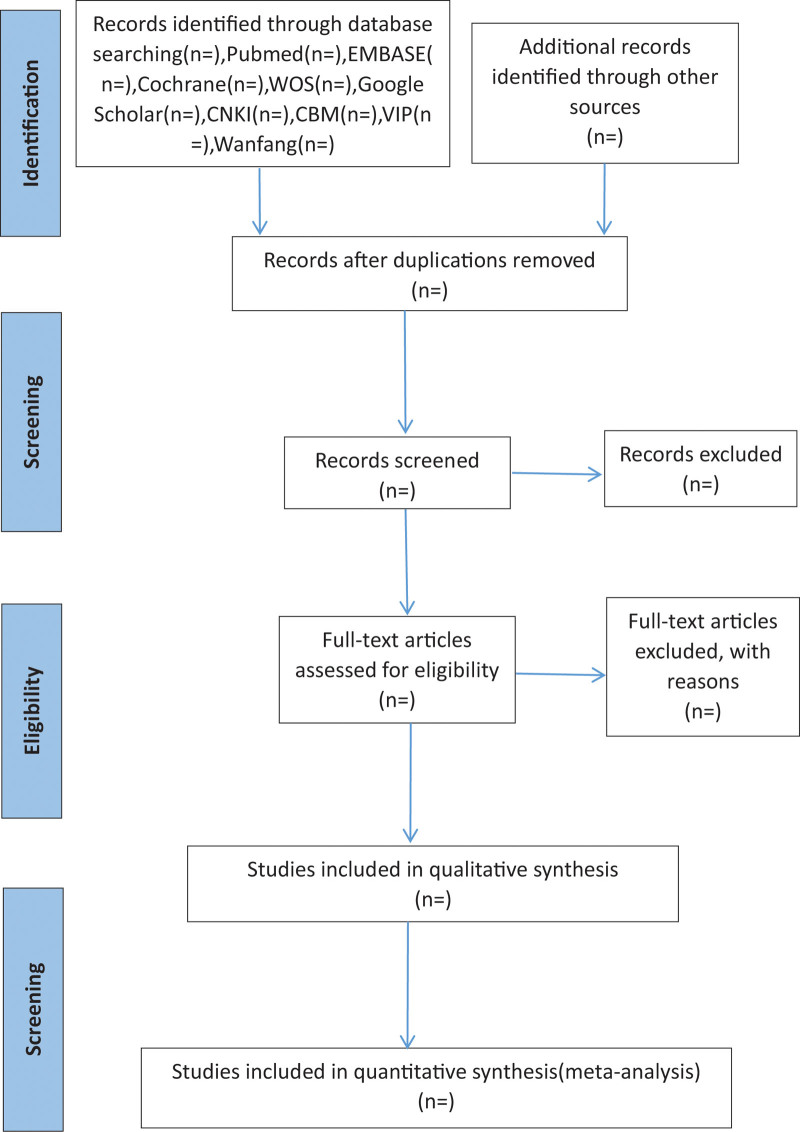
Flow chart of this systematic review.

The literature search and screening will be carried out independently by 2 researchers. First, an initial screening will be performed based on the title, abstract and subject terms, and duplicate literature will be excluded. Then, the literature that meets the requirements is screened by reading the full text. If disagreements exist, they should be further discussed or referred to a third researcher for evaluation. EndNote software is used for literature management. In addition, the reasons for exclusion of literature will be recorded in the corresponding study. The literature screening process used in this study is shown in Figure 1.

#### 2.3.2. Data extraction and management.

Two authors will independently generate a table for data extraction. The table will include general information (title, authors’ names, funding status, study country, publication date); study details (study objectives, sample size, inclusion and exclusion criteria, interventions, outcome indicators, randomization, blinding, selective reporting, adverse effects); and subject profile (gender, age, duration of disease, ethnicity, TCM evidence of disease). If there are discrepancies in the results of the extracted data, they will be discussed or submitted to a third uninvestigator for adjudication. If the required data are lacking, the authors of the article will be contacted to obtain relevant information, and if data are still not available, studies with missing data will be excluded.

#### 2.3.3. Assessment of risk of bias in included studies.

The 2 authors will independently assess the quality of the included studies separately using the methods recommended in the Cochrane Handbook for Systematic Reviews of Interventions.^[29]^ The following risk of bias domains will be assessed: sequence generation (selection bias); allocation concealment (selection bias); blinding of participants and personnel (performance bias); blinding of outcome assessment (detection bias); incomplete outcome data (attrition bias); selective outcome reporting (reporting bias) and other bias. We will attempt to describe what is reported to have happened in each study for each domain of risk of bias. Thus, we will be able to provide the rationale for the judgement of whether this domain is at low, high or unclear risk of bias. Where necessary, we will contact authors of included studies for missing information or clarification. If all domains are at low risk of bias, the overall risk of bias of individual studies will be categorized as low risk of bias. Otherwise, overall risk of bias will be categorized as high risk of bias.^[30]^ The “risk of bias” summary will be presented graphically.

#### 2.3.4. Data analysis and synthesis.

Data analysis will be performed using the Review Manager V.5.3 software provided by Cochrane Collaboration (www.cochrane.org). The *Q* test and *I*^2^ statistic will be used to assess the heterogeneity of the included studies.^[31]^ The relative risk will be used to analyze dichotomous risk. The fixed-effects model will be used to combine the data if the statistical heterogeneity is low (*P* ≥ .1 and *I*^2^ ≤ 25%), or a random-effects model will be used if the statistical heterogeneity is high (*P* < .1 and *I*^2^ > 50%). The mean difference with 95% confidence interval will be used for the continuous variables, and standardized mean difference and 95% confidence interval will be used for the continuous variables if the units are different.

#### 2.3.5. Subgroup analysis.

When sufficient data are available, we will perform subgroup analysis to explore heterogeneity. We will perform subgroup analysis according to age, gender, TCM evidence type of COVID-19, different stages of COVID-19, Chinese herbal medicine name, Chinese herbal medicine dose, dosing time and type of control group (excluding Chinese herbal medicine prescriptions for qi and blood supplementation, western medicine treatment, western and Chinese external treatment methods, conventional or standard care, placebo or no treatment, etc.).

#### 2.3.6. Sensitivity analysis.

This study will carry out sensitivity analysis by changing the effect indicators and statistical model, and deleting each included study one by one to verify the stability of the study results. If different conclusions are reached, the results of the meta-analysis are carefully obtained by discussion between the 2 authors or by evaluation by a third person.

#### 2.3.7. Assessment of reporting bias.

When a meta-analysis includes 10 or more RCTs, we will assess asymmetry using funnel plots visually.^[30]^

### 2.4. Patient and public involvement

Patients or members of the public were not involved in the design of this study.

### 2.5. Ethics and dissemination

Ethical approval and consent are unnecessary because no primary data will be collected. The results will be disseminated through peer-reviewed publications.

## 3. Discussion

The COVID-19 pandemic has been ongoing for nearly 3 years, severely affecting the economies of various countries and causing profound disasters worldwide, especially for people in areas with low levels of medical care. Studies have confirmed that the development of New Crown pneumonia is closely related to viral infection of the lungs and autoimmune deficiency, and leads to numerous complications through related mechanisms.^[5,32–34]^ In China, the combination of Chinese and Western medicine has become the highlight of the fight against the epidemic with good results, while Chinese medicine is gradually being accepted overseas as the epidemic continues to spread.^[20,21]^ Improving immunity has traditionally been one of the principles adopted by Chinese medicine when facing infectious diseases. One of the principles, in addition, the direct or indirect antiviral and anti-inflammatory effects of TCM are being explored and promoted.^[10,35–37]^ In the New Crown epidemic, the “3 drugs tripartite” consisting of Lianhua Qingwen Capsule, Jinhua Qinggan Granule, Xuebijing Injection, Lung Cleansing and Detoxifying Decoction, Huashibaidu Formula, and Xuanfeibaidu Granule, summarized or promoted with Many herbal formulas represented by classical TCM formulas such as Yupingfeng powder, Shengmai power and Dushen decoction contain herbal medicines such as Huangqi, Renshen, Dangshen, Xiyangshen, Baizhu, Shanyao, Gancao, Danggui, Shudi, Hongjingtian, Baishao, Gouqi, Dazao.^[38]^ Many of these herbal medicines are not only used throughout but are especially important in the recovery period.^[19,39]^

SARS-CoV-2 enters the body by binding to angiotensin-converting enzyme-2 (ACE-2), a receptor, and acts by initiating the S protein (TMPRSS2) on this virus in the presence of the transmembrane protein serine-2.^[40–42]^ Because ACE-2 is widely expressed in numerous respiratory, neurological, digestive, urinary, cardiovascular, lymphatic, and other systems,^[43]^ it is easy to understand why so many complications accompany patients with neocoronary artery disease. Glycyrrhizin, the main active component of licorice, has been shown to interact directly with ACE2, thereby blocking the binding of SARS-CoV-2 to ACE-2, and thus licorice may be a potential drug for the treatment of COVID-19.^[44,45]^ In addition, licorice has better anti-inflammatory, antiviral, antibacterial, immunomodulatory, and anti-pulmonary fibrosis advantages.^[46]^ This further reflects the fact that licorice is used in the treatment of neocon of the importance and necessity of its presence in almost all prescriptions. Studies have confirmed that in SARS-CoV-2 infection, helper T cells, which are a subpopulation of CD4^+^ T lymphocytes, secrete significantly more for example, interleukins IL-4 and IL-10, which leads to a restricted inflammatory response and immune function, resulting in severe lung injury.^[47]^ Yam polysaccharide, the active ingredient of yam, induces humoral immunity and Th1/Th2 immune responses, enhanced activation of CD4+ and CD8+ T lymphocyte subsets, and promoted activation of cytotoxic T lymphocyte responses.^[48]^ After SARS-CoV infection, dysregulation of responses to numerous proinflammatory cytokines (e.g., IL-6, IL-8, IL-1β, and tumor necrosis factor-α [TNF-α]) and chemokines (e.g., CCL-2, CCL-3, CCL-5, IP-10) due to leading to a storm of inflammatory cytokines that exacerbate dyspnea and increase mortality.^[49,50]^ The development of acute respiratory distress syndrome is inextricably linked to increased endothelial and epithelial permeability of the lung, in addition to excessive inflammation.^[51]^ Studies have confirmed that astragalus not only inhibited some pro-inflammatory factors such as IL-6 and TNF-α reduced the threat posed by acute respiratory distress syndrome, the leading cause of death in COVID-19, and effectively inhibited viral entry and replication.^[52]^ Previous studies have shown that flavonoids regulate the expression and activation of cytokines such as IL-1β, TNF-α, IL-6, and IL-8, as well as many proinflammatory factors such as nuclear factor kappa-light chain enhancer, which activates B cells, and activator protein-1 gene expression,^[53]^ and quercetin, an important component of flavonoids, exerts a synergistic antiviral effect when co-administered with vitamin C to improve the treatment of COVID-19.^[54]^ And there is network pharmacology and molecular docking showing that quercetin binds well to ACE2 to block the entry of SARS-CoV-2 and can bind to the RBD structure of the S protein on this virus to S protein’s RBD structure to act as a viral neutralizer to reduce toxicity.^[55]^ And quercetin is widely found in Chinese herbal medicines such as angelica, peony, and ginseng. This virus can cause cardiovascular lesions due to atherosclerosis, and studies have confirmed that angelica regulates blood lipids, serum LDL, blood glucose, and serum cholesterol significantly.^[56]^ SARS-CoV-2 is an RNA virus that is unstable and also prone to mutation, posing a great challenge to clinical treatment.^[32]^ Some animal experiments have shown that related qi-supplementing herbs can regulate inflammatory cytokine expression by infecting mRNA expression of inflammatory cytokine imbalance in influenza virus organisms, thereby resisting immune inflammatory damage in the lung.^[57]^ Fatigue, as one of the main manifestations of COVID-19, poses a challenge to its treatment, and previous studies have demonstrated that herbs such as Astragalus, Ginseng, Rhodiola, and Schisandra can enhance and regulate mitochondrial function, improve glycogen storage, reduce the accumulation of metabolites, enhance Anti-fatigue effects have also been demonstrated in animal experiments.^[58]^ Animal experiments have also shown that LBP, a chemical component of Lycium barbarum, achieves anti-fatigue effects by regulating oxidative stress and energy metabolism in rats.^[59]^ This provides a relevant theoretical basis for the treatment of clinical fatigue symptoms in COVID-19 with qi-supplementing herbs.

COVID-19 belongs to the “epidemic disease” category in Chinese medicine. The epidemic enters through the nose and mouth, first invading the lungs and other organs of the respiratory system, and over time, through the relationship of mutual restriction, over-restriction, and counter-restriction of 5 phases, leading to or aggravating other internal organs such as the heart, spleen, stomach, liver, and kidney, etc., and this series of production is inseparable from the weakness of the body’s vital energy. In the classical works of Chinese medicine, it has long been written that “when the vital energy exists within, the pathogenic qi cannot dry up; where the pathogenic qi comes together, its vitality will be deficient, “ a clinically proven axiom, the “ vital energy “ here refers to what modern medicine calls immunity. Therefore, in the treatment of this disease, Chinese medicine focuses on protecting the body’s vital energy, and the main source of improving the body’s vital energy is the use of herbal medicines that benefit qi and blood.

## 4. Conclusion

This systematic evaluation and meta-analysis will assess the efficacy and safety of qi-supplementing and blood-supplementing herbs in patients with COVID-19. The evaluation reveals that, in a broad sense, tonifying Qi and Blood herbal medicines have dual effects on COVID-19 in terms of antiviral and anti-inflammatory effects and improving human immunity, and these herbal medicines play an important role at different times of the disease and throughout, and with the continuing pandemic of new crowns, herbal medicines, especially tonifying Qi and Blood herbal medicines, are increasingly valued.

## Author contributions

FY and XZ contributed equally to this study. FY conceived the study and developed the first framework of the manuscript. FY and XZ drafted the manuscript, and ZZ and HL were involved in the development of the search strategy. JL and NB will read the full text of the study and extract the data and perform data synthesis. FY and XZ assessed the quality of the included studies. If there is any disagreement, NG will arbitrate. The manuscript was revised by NG. All the authors read and approved the final manuscript.

**Conceptualization:** Feifei Yang, Xiaosi Zhang.

**Data curation:** Naizhi Geng.

**Formal analysis:** Zhe Zhang, Hao Lu, Ning Bai.

**Funding acquisition:** Naizhi Geng.

**Methodology:** Zhe Zhang.

**Software:** Hao Lu, Jiawei Li.

**Visualization:** Naizhi Geng.

**Writing – original draft:** Feifei Yang, Xiaosi Zhang.

**Writing – review & editing:** Feifei Yang, Xiaosi Zhang.
